# Insulinoma presenting as refractory seizure disorder

**DOI:** 10.12688/f1000research.1-15.v1

**Published:** 2012-09-21

**Authors:** Pamela Correia, Roopal Panchani, Rajeev Ranjan, Chandrashekhar Agrawal

**Affiliations:** 1 Department of Neurology, Sir Ganga Ram Hospital, New Delhi, India

## Abstract

Hypoglycaemia can lead to acute disorders of cognition, consciousness, epilepsy, transient ischemia, psychosis and chronic disorders of dementia and neuropathy. Misdiagnosis and delay in treatment are common and prolonged hypoglycemia can lead to permanent neurological deficit or fatal coma. Hypoglycemia caused by an insulinoma is a readily treatable condition that should be considered in the differential diagnosis of intractable seizures. The following case report highlights the need for careful reassessment of all seizures that are atypical and refractory to medication.

## Case report

A 29 year old man had a one year history of multiple seizure episodes, which were as frequent as 3–4 episodes in a week despite being on regular antiepileptic medication of 3 drugs including carbamazepine, sodium valproate and clobazam. All the episodes were uncannily similar in that they always occurred on awakening and in the early hours of morning, between 5.00 to 7.00 am. The attacks were episodic stereotyped confusional spells characterized by abnormal posturing, perioral and eyelid twitching and unresponsiveness. The attacks would usually last from a few minutes to about half an hour. Upon initial OPD visits, compliance was checked and metabolic causes were ruled out. MRI Brain was done which was normal. No interracial epileptic activity was observed during prolonged video EEG monitoring.

**Figure 1.**
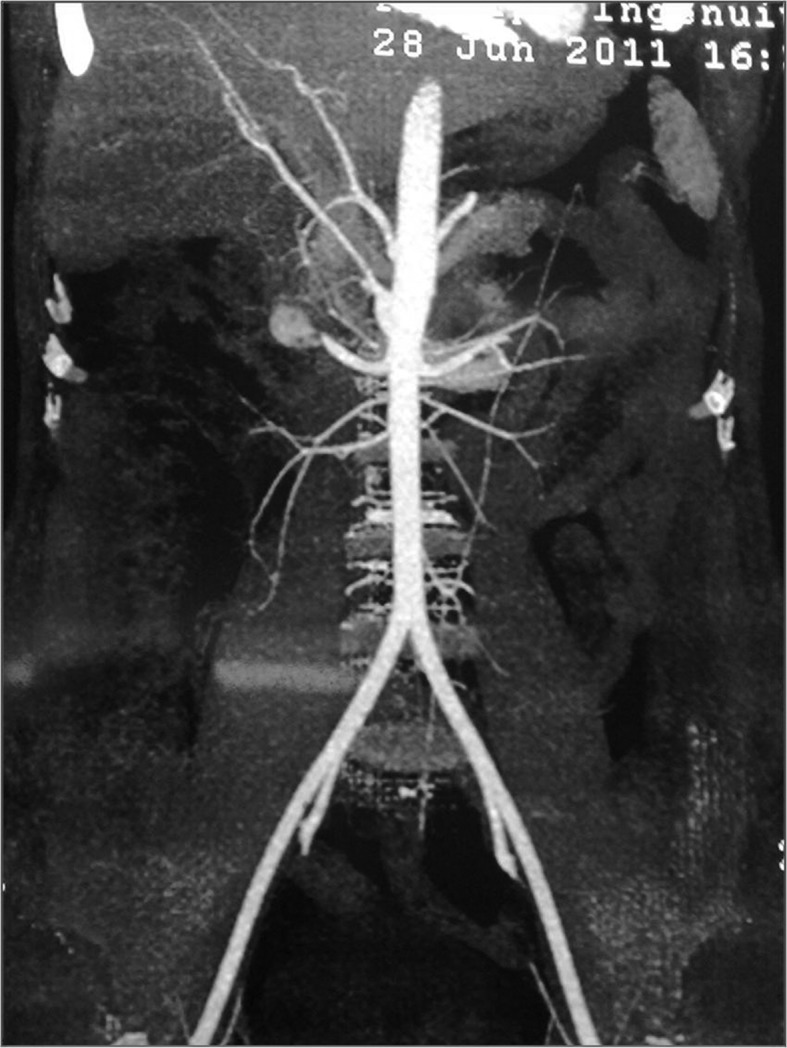
Contrast enhanced CT revealed a solitary insulinoma in the head of pancreas.

**Figure 2.**
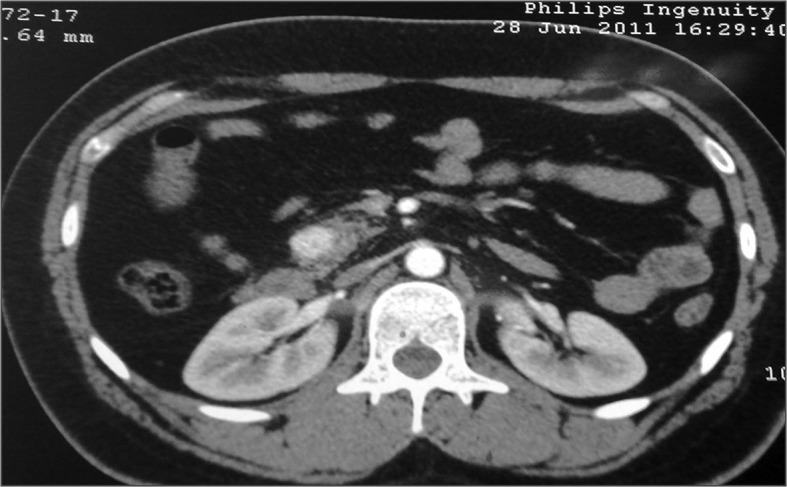
Contrast enhanced CT (transverse section) revealed a solitary insulinoma in the head of pancreas.

Five months later, seizures started occurring at increased frequency. On presentation to our hospital he was brought early morning in an unconscious state and was found to have hypoglycemia (48 mg/dl) which recovered immediately after treatment. On further questioning, it was apparent that his previous seizures tended to occur in early morning or several hours after the meal and that the post seizure confusion state could be shortened if he ate something during the confusion period. The patient had gained 10 kg weight over the period of last 6 months. The subsequent endocrine evaluation suggested insulinoma. Contrast enhanced CT revealed a solitary insulinoma in the head of pancreas. ( Figure 1 and Figure 2) The patient underwent a surgical removal of the insulinoma with a Whipple’s resection procedure. A benign insulinoma was confirmed on histopathological examination. Following the procedure he has been totally seizure free and asymptomatic for last 6 months.

## Discussion

Insulinomas are the commonest hormone-secreting tumor of the gastrointestinal tract; the incidence is 4 cases/million/year. Tumors may occur as a unifocal sporadic event or in 5–10% of patients with MEN-1. 10% are metastatic, and a further 10% are multiple but behave as benign tumors. Insulinomas are found throughout the pancreas and are small (usually 10–20 mm). The median interval from onset of symptoms to the diagnosis of insulinoma is 2 years with a wide range of one month to 30 years as reported by Service F. and collegues ^1^. Presentation is usually insidious with neuroglycopenia and fasting hypoglycemia.

Diagnosis relies on key neuroglycopenic and sympathetic symptoms together with biochemical confirmation by documenting blood glucose levels below 50 mg/dl during monitored symptomatic episodes (that improve with oral intake), elevated C-peptide levels (>200 pmol/L), increased serum insulin levels (>5 µmol/mL), and absence of plasma sulfonylurea.

Many patients with an insulinoma do not report the adrenergic symptoms of hypoglycemia and present with neurological or psychiatric manifestations that often lead to misdiagnosis. The delay in diagnosis is due to several factors. Firstly, the symptoms of insulinoma lack specificity, including various seizure disorders, personality change, bizarre behavior, amnesia, convulsions, and incidentally dystonia and polyneuropathy; these symptoms are similar with many common neurological and psychiatric disorders. Secondly, fasting blood glucose level can be normal in some patients. Thirdly, hypoglycemia itself induces neuroglycopenic and autonomic unawareness.

Seizure disorder has been described in few contemporary cases of persistent hypoglycemia later on diagnosed as insulinoma two of them reported by Akanji A. and colleagues in 1992 followed by Basil C. and Pack A. in 2001 ^2, 3^. Of late Wang S. and his colleagues described recurrent episodes of automatism, confusion and convulsion with electroencephalography (EEG) findings resembling the pattern in complex partial seizures in a case of insulinoma ^4^. Jaladyan V. and Darbiyan V. described a case of a girl presenting with drug-refractory myoclonia and generalized tonic-clonic seizure (GTCS) initially misdiagnosed as having juvenile myoclonus epilepsy (JME) before insulinoma was detected ^5^. O’sullivan S. and Redmond J. also presented a case of insulinoma misdiagnosed as late onset refractory epilepsy ^6^.

As in our case, the interpretation of clinical symptoms and normal EEG with seizures refractory to treatment was indicative of any toxic or metabolic cause or poor compliance to treatment. The possibility of hypoglycemia was excluded by three normal blood glucose measurements. Furthermore hypoglycemia due to an insulinoma especially in a young age group comes little lower in the list of other possible causes. Insulinoma, responsible for profound hypoglycemia usually has its onset in middle or older age. Symptoms are usually prominent following prolonged fasting states, especially in the morning as in our patient, but also in late afternoon.

Insulinoma can also mimic other neurological diagnoses apart from seizures. In Daggett and Nabarro's review of 252 reported cases the most common neurological symptoms were confusion, coma, and seizures ^7^. Non-specific headache, generalized weakness, paraesthesia, and stroke-like paralysis were less frequent and dizziness and dysarthria were uncommon. Newman and Kinkel reported a diabetic woman who developed two episodes of limb choreoathetosis and opisthotonus associated with hypoglycemia ^8^. Winer *et al.* described a woman with an insulinoma who, when recovering from hypoglycemic attacks developed abnormal posturing of her body ^9^. Chronic neuropathic and dementing syndromes due to hypoglycemia have also been described by Danta G. and Snook J. and his colleagues ^10, 11^. In a prospective survey done by Harrington M., two of 25 patients referred to neurologists with "funny turns" were found to have an insulinoma ^12^.

This case highlights the importance of considering hypoglycemia in atypical neurological or psychiatric episodes and emphasizes the role of inpatient evaluation of refractory epilepsy. Neuroglycopenia should be considered in all patients with seizures, and other neuropsychiatric symptoms, especially if they do not conform to diagnostic criteria or respond to standard treatment. Taking a full history (including relationship of attacks to food, non-stereotyped or atypical attacks, and poor response to treatment) and clinical suspicion are the key to making a diagnosis of insulinoma. Once suspected, confirming the diagnosis with a 72 h fast is relatively simple.

Benign insulin secreting adenomas are a potentially curable cause of seizures but may be fatal if unrecognized. Our case reiterates the importance of evaluating the metabolic cause of refractory seizure disorders. Although sometimes insulinoma shares some common semiological and EEG features, the possibility of atypical causes like insulinoma associated seizures should be considered in patients with some clues to diagnosis like close relationship to food intake, history of weight gain atypical attacks, seizures refractory to treatment. Seizures that present with such a particular prototypical pattern should arouse a strong clinical suspicion. Hence, early diagnosis and treatment can free many a patient from burdensome multiple antiepileptic treatments.

## Consent

Written informed consent was obtained for publication of their clinical details and images was obtained from the patient.
