# Supplementary material for: Insulinoma presenting as refractory seizure disorder
Source: F1000Res. 2012 Sep 21;1:15. [Version 1] doi: 10.12688/f1000research.1-15.v1 (PMC3790603; doi:10.12688/f1000research.1-15.v1)
Supplement: Peer review history

# Peer review: Referee response for version 1

**Published:** 2012-10-02

**Authors:** Niki Karavitaki

**Affiliations:** 1 Oxford Centre for Diabetes, Endocrinology and Metabolism, Churchill Hospital, Oxford, UK

I have read this submission. I believe that I have an appropriate level of expertise to confirm that it is of an acceptable scientific standard.

# Peer review: Referee response for version 1

**Published:** 2012-09-29

**Authors:** Francesco Minuto

**Affiliations:** 1 Department of Endocrinology and Metabolism, University of Genova, Genova, Italy

On viewing this seems like properly done research. The work itself is not really uncommon, neither new but it is worth publishing.

I have read this submission. I believe that I have an appropriate level of expertise to confirm that it is of an acceptable scientific standard.
